# Decoding the Role of Temperature in RNA Virus Infections

**DOI:** 10.1128/mbio.02021-22

**Published:** 2022-08-18

**Authors:** Karishma Bisht, Aartjan J. W. te Velthuis

**Affiliations:** a Department of Molecular Biology, Princeton University, Princeton, New Jersey, USA; Ohio State University

**Keywords:** RNA virus, RNA polymerase, transmission, replication, zoonotic, stability, innate immune response, flavivirus, influenza A virus, temperature, SARS coronavirus 2, respiratory virus, arbovirus

## Abstract

RNA viruses include respiratory viruses, such as coronaviruses and influenza viruses, as well as vector-borne viruses, like dengue and West Nile virus. RNA viruses like these encounter various environments when they copy themselves and spread from cell to cell or host to host. *Ex vivo* differences, such as geographical location and humidity, affect their stability and transmission, while *in vivo* differences, such as pH and host gene expression, impact viral receptor binding, viral replication, and the host immune response against the viral infection. A critical factor affecting RNA viruses both *ex vivo* and *in vivo*, and defining the outcome of viral infections and the direction of viral evolution, is temperature. In this minireview, we discuss the impact of temperature on viral replication, stability, transmission, and adaptation, as well as the host innate immune response. Improving our understanding of how RNA viruses function, survive, and spread at different temperatures will improve our models of viral replication and transmission risk analyses.

## INTRODUCTION

RNA viruses are intracellular pathogens that cause frequent epidemics and occasional pandemics. Of particular importance are infections with respiratory and vector-borne RNA viruses, such as influenza A virus (IAV), Zika virus (ZIKV), and West Nile virus (WNV) (1, 2). In addition, humans are frequently exposed to RNA viruses that spill over from reservoir hosts, including rodents, bats, and wild birds (3, 4). Some of these emerging viruses, which include the Ebola virus (5), the 1918 pandemic IAV (6), and severe acute respiratory coronavirus 2 (SARS-CoV-2) (7, 8), have caused devastating epidemics and pandemics in recent history.

It is likely that RNA virus spillover will continue to happen in the future (3), creating new zoonotic events in which RNA viruses will encounter human physiology for the first time. In particular, IAVs have a strong zoonotic potential, and transmission of various avian subtypes, such as H5N1, H5N8, and H7N1, to humans has been reported for decades (9). Moreover, different influenza virus types, such as IBV, ICV, and IDV, as well as a plethora of IAV subtypes circulate in bats (10), horses (11), swine (12), and cattle and goats (13), providing plenty of genetic diversity to support zoonotic events. In addition, sequencing of environmental samples suggests that other hosts, like the Wuhan Asiatic toads, Wenling hagfish, and spiny eels (14, 15), may harbor other influenza virus types that could potentially be responsible for a zoonotic event in the future. Other important zoonotic RNA viruses that spill over from bats or other mammals to humans include SARS-CoV, Middle East respiratory syndrome CoV (MERS-CoV), and SARS-CoV-2 (16), as well as the paramyxoviruses Nipah virus and Hendra virus (17, 18).

**FIG 1:**
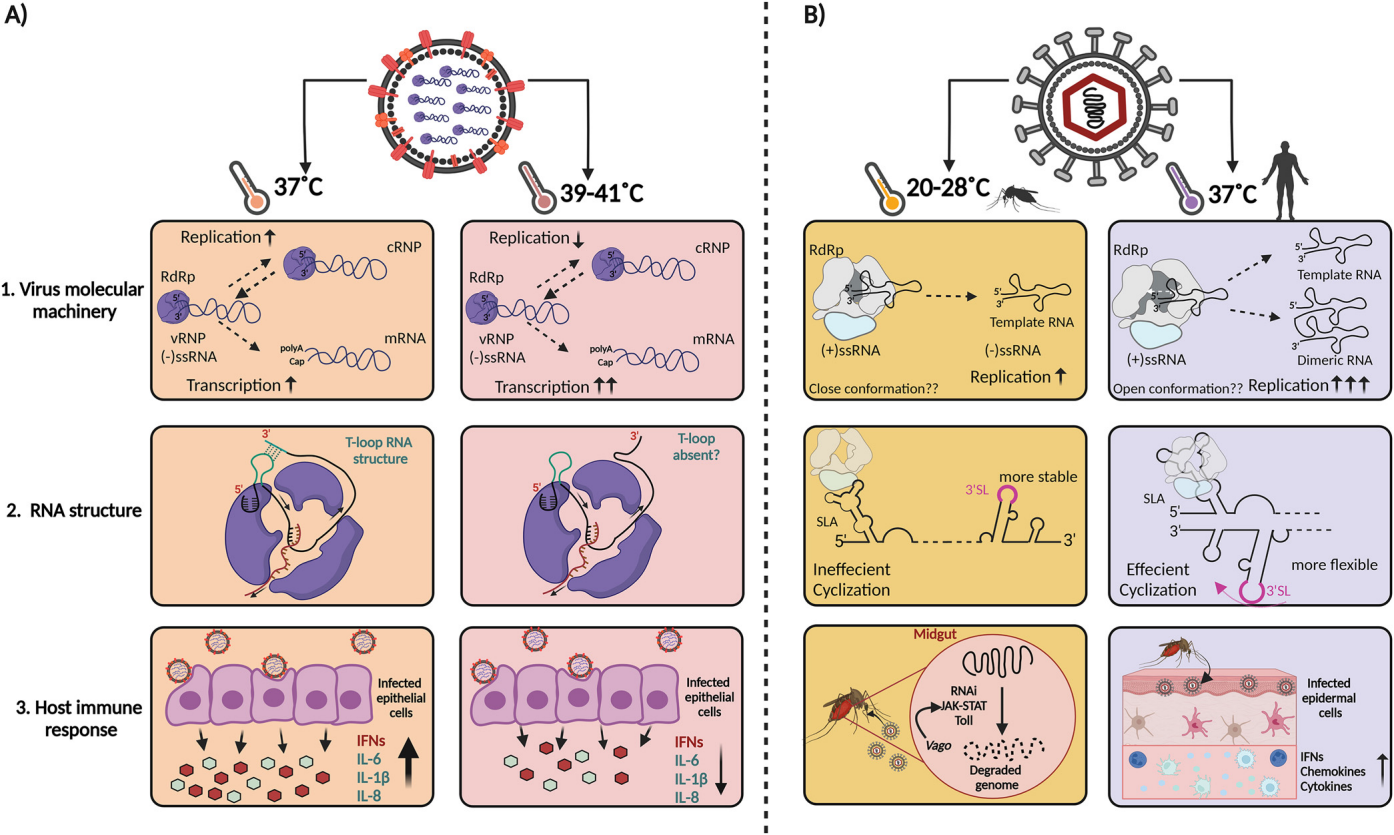
The effect of temperature on viral RNA synthesis, RNA secondary structure, and host immune responses. (A) The RNA-dependent RNA polymerase (RdRp) of IAV is influenced by temperature fluctuations, resulting in a higher viral replication rate at 37°C than at 40°C and an increased transcriptional activity. Temperature changes also affect secondary RNA structures. When present, template loops (t-loop) can induce RdRp stalling and the activation of the innate immune response. However, at higher temperatures, base pairing in the structures is reduced, increasing viral RNA synthesis and preventing innate immune activation. (B) Arboviruses can infect both insects (ectotherms) and humans (endotherms). Arbovirus RdRps can be influenced by thermal fluctuations, resulting in different rates of initiation and the formation of either single full-length copies or copy-backs that are double the expected size. Additionally, temperature impacts the transition of the viral genome from a linear to a circular form. The higher flexibility of the 3′ stem-loop structure (3′ SL) at a higher temperature allows sequences in the two-genome termini to hybridize. In turn, circularization of the genome assists the viral RdRp to efficiently bind to the 5′ end of the genome (5′ SLA) and transfer to the 3′ terminus to initiate viral RNA synthesis. Lastly, the host immune response differs dramatically in mosquito and mammalian hosts. In the case of mosquitoes, the Toll signaling pathway, RNAi, and JAK-STAT pathways are activated upon arbovirus infection at the right body temperature, while in a mammalian host, cytokines and interferon responses are elicited by an arbovirus infection. The figure was created using BioRender.

In birds, respiratory RNA viruses replicate at 42°C (19), in bats up to 41°C (10, 20), and in cold-blooded fish and frogs at ambient temperatures (21). These temperatures are different from the human respiratory system, which starts at 25 to 34°C in the nasal mucosa, increases to 34 to 35°C in the trachea and bronchi, and finally reaches 37°C in the alveoli (22–24). In line with the environmental conditions of the human respiratory tract, *in vitro* human-adapted respiratory RNA viruses, such as IAV, CoV, rhinovirus (RV) and respiratory syncytial virus (RSV), grow best at temperatures that match the human upper respiratory tract (Fig. 1A), i.e., 32 to 33°C (25, 26). In contrast, spillover viruses grow better at the higher temperatures of their reservoir hosts (27, 28).

Temperature also has a profound effect on the survival and transmission of vector-borne viruses, such as ZIKV, dengue virus (DENV), and chikungunya virus (CHIKV). These viruses are transmitted through an intermediate host, such as a mosquito or tick. In contrast to mammals, mosquitoes and ticks are ectotherms whose physiology and survival are strongly dependent on the temperature of the environment (29, 30). Consequently, vector-borne viruses need to infect and replicate at temperatures that can be far different from their optimal replication temperature (31, 32), which can have a profound effect on their infection cycle and transmission efficiency (33). Moreover, as our climate changes, and insects migrate to and survive in areas where they previously could not, vector-borne diseases are now being detected in countries where they were previously not present (34, 35). What this means for human disease in the future is still only partially explored.

Many studies have explored the molecular mechanisms through which bacteria and bacterial diseases are affected by temperature (36–39), but how temperature affects RNA virus replication, modulates the immune response, and influences virus survival outside the host are not fully understood. These are all key aspects in the viral infection cycle. Following binding to host cell receptors, RNA viruses are replicated by a viral RNA-dependent RNA polymerase (RdRp) in the cytoplasm or nucleus of the host cell (40, 41). Here, the viral RdRp interacts with one or more host factors to copy or transcribe the viral RNA genome, as discussed in several reviews on positive-sense (42–44) and negative-sense (45–47) RNA viruses. Next, viral mRNAs are translated and viral proteins assemble together with viral RNA into new virions. The temperature at which RNA synthesis and virion assembly take place impacts the folding of RNA secondary structures in the genome and protein-RNA and protein-protein interactions, as well as the conformation and activity of viral enzymes (48–52). The temperature at the time of the infection also affects the innate immune response triggered by the infection and production of viral RNA molecules (53–55) and thereby viral growth and the transmission efficiency. Knowledge of the impact of temperature on viral RNA synthesis, the immune response, and the stability of RNA viruses in different environments is important for our understanding of their transmissibility and the design of antiviral strategies, as well as for estimating the likelihood that humans may contract spillover RNA viruses in the future.

## TEMPERATURE AFFECTS VIRUS TRANSMISSION

### Respiratory RNA viruses.

Human-adapted respiratory viruses typically replicate in the upper respiratory tract and cause disease in seasonal epidemics, typically in the autumn or winter in the Northern and Southern hemispheres and after the rainy season in tropical climates (56, 57). In contrast, emerging respiratory viruses can spill over at any time and impact human health in epidemics as well as pandemics that are not bound to specific seasons. Spillover from reservoir animals can also take more different forms, including aerosol transmission via the fecal-respiratory route (58–60). For both human-adapted and zoonotic viruses, the stability and transmissibility at different temperatures and the relative humidity at a particular temperature play key roles in viral spread and epidemiology.

RNA virus stability and transmissibility are dependent on many variables. Human-to-human transmission of respiratory viruses occurs through aerosols (particles <100 μm in diameter), droplets (particles 100 to 500 μm in diameter), or fomites (droplets on surfaces) created during sneezing, coughing, singing, or speech or the deposition of respiratory droplets to a surface via touch (56, 58–60). In general, aerosol-sized droplets originate in the lower respiratory tract and laryngeal region, while larger droplets are produced in the nasal cavity (61). Once airborne, an aerosol starts to evaporate at a rate that is a function of its diameter and the physical properties of its environment, like the local humidity, airflow, and temperature (59). As a result, aerosols and droplets become smaller once they are exposed to the environment, enabling airborne particles to spread further. However, because aerosols and droplets can emerge from various parts of the respiratory tract and viruses replicate with different efficiencies in the different parts of the respiratory tract, the viral content, surfactant protein content, and salt content will vary equally among droplets, making it harder to generalize how different environmental variables impact respiratory virus spread (59).

In addition to the size and spread of airborne particles that carry RNA viruses, the stability of RNA viruses inside these particles is a critical factor for transmission. In laboratory air, half of the SARS-CoV-1 and SARS-CoV-2 virions emitted remain infectious for just over an hour, whereas in fomites, these viruses can be stable for several hours to days, depending on the surface (62). However, differences in stability have been observed among laboratory-grown SARS-CoV-2 variants (63), indicating how mutations in viral proteins modulate a virus’s vulnerability to the environment. Various studies have been performed to quantify the impact of temperature and humidity on virus stability and transmission. IAV airborne transmission via droplets was effective to modestly efficient at up to 65% humidity, but transmission failed at 80% humidity (64). Airborne transmission also proved to be efficient at 5°C and 20°C, but transmission was inhibited at the relatively high temperature of 30°C (64). In contrast, short-range aerosol transmission, which minimizes the exposure of a virus to the environment, is equally effective at 20°C and 30°C (65). A similar trend has been observed for SARS-CoV-1 transmission (66) and RV transmission (67).

The above observations are supported by studies showing that exposure of SARS-CoV-2 virus-like particles (VLPs) to 34°C induced structural degradation of the VLPs, while exposure of VLPs to 22°C did not (68). Moreover, nuclear magnetic resonance (NMR) analysis of an intact IAV lipid membrane showed that viral membranes are in a disordered state at 37°C and above but transition to liquid-ordered and solid-ordered states when the temperature drops below 34°C and 10°C, respectively, showing that lower environmental temperatures promote lipid ordering and providing a mechanism for the stability of respiratory viruses at lower temperatures (69). Together, these studies indicate that respiratory viruses spread best at low temperatures, as this keeps viruses stable, and at low humidity, as this reduces aerosol and droplet sizes and enhances transmission distances. Spread at high temperatures does occur, provided that exposure to the environment is minimized as the positive effect of temperature on transmission distance is outweighed by its negative effect on virus stability.

### Vector-borne viruses.

Viruses whose infection cycle involves vertebrate and invertebrate hosts need to replicate at different body temperatures. For instance, arboviruses, such as flaviviruses and alphaviruses, infect both insects, which are ectotherms, and vertebrate hosts, which have a body temperature of 37°C or higher. Because the replication cycle of these viruses involves ectotherms, this means that the temperature of the environment has a substantial impact on viral infection and transmission. But the environmental temperature does not only affect insect biology, survival, and spread: it also impacts arbovirus transmission (31, 70, 71). Models estimating the effect of temperature on the ZIKV transmission risk showed that the optimal transmission temperature ranges from 24 to 29°C (71, 72). At lower temperatures, such as 16 to 20°C, ZIKV transmission drops steadily, likely because viral dissemination from the midgut and invasion of the salivary gland are reduced under these conditions. At higher temperatures (e.g., 38°C), mosquitoes are efficiently infected, and the virus disseminates to the salivary gland. However, the high mortality rate among mosquitoes at such temperatures limits mosquito-human interactions and thus the ability of the virus to transmit (71). Similar effects of temperature on transmission have been observed for other vector-borne viruses. For DENV, a wide optimal temperature range, 24 to 33°C, was reported (73), while for WNV, Rift Valley fever virus (RVFV), and Ross River virus, more narrow optimal transmission temperatures, 23°C, 26°C, and 26°C, were found, respectively (74, 75).

The optimal transmission temperature depends on many factors, and the relation between temperature and virus transmission can be confounded by the vector species studied, since the heat tolerance, gonotrophic period, and feeding behavior can vary among mosquitos and other insects (76). Moreover, humidity and the seasons can also impact the findings, as well as the movement and distribution of other host animals (77). In addition, temperature affects the incubation period of the virus in a strain-specific manner, as illustrated with different WNV strains, and it may modulate the stability of components of vector-borne viruses (78, 79). Indeed, cryo-electron microscopy (cryo-EM) imaging of DENV particles showed that these virions undergo conformational changes at 37°C (80, 81). However, these changes did not affect the infectivity of the virions, suggesting that the temperature-dependent restriction occurs downstream of infection. We discuss the effect of temperature on viral RNA synthesis below.

## TEMPERATURE IMPACT ON VIRUS REPLICATION, TRANSCRIPTION, AND RNA STRUCTURE

### Influenza viruses.

Human-adapted respiratory RNA viruses, such as seasonal IAV H1N1, typically infect epithelial and immune cells in the upper respiratory tract, where the temperature is around 31 to 33°C. Emerging respiratory RNA viruses, such as avian IAV H5N1, frequently infect the lower respiratory tract, where the temperature is 37°C and closer to the temperature of the avian gut, where IAVs grow at 41°C. Studies comparing the growth rates of IAVs found that avian IAVs grow faster and to higher titers at 37°C than at 33°C, whereas human-adapted IAV strains grow at similar rates and to similar titers at both temperatures (82–85). Interestingly, an IAV isolate from the 1918 pandemic grew better at 37°C and 41°C than at 33°C, in line with the preference of emerging IAV to replicate in the lower respiratory tract (85) (note that other factors, such as sialic acid receptor binding, contribute to the preference for the lower respiratory tract as well [86]). Interestingly, differences in temperature sensitivity vary among influenza viruses that spillover from other mammals. IDV isolated from cattle showed reduced viral growth at 37°C compared to that at 33°C on differentiated human airway epithelial cells (hAECs) and other respiratory cells (84, 87), while an IDV isolated from swine grew to similar levels at 37°C and 33°C (84). Also, swine IAV isolates showed different temperature sensitivities, with isolates from 1930 and 1982 displaying reduced viral growth at 33°C and a 1998 isolate showing preferential growth at 33°C (27, 85). Since avian, swine, and human IAVs frequently reassort in swine, supported by the segmented IAV genome, it is likely that the gene constellation, and in particular the constellation of the RdRp subunits, at the time of isolation affected the observed viral growth rates at different temperatures *in vitro*.

The IAV RdRp consists of three subunits, PB1, PB2, and PA, and replicates the viral RNA (vRNA) via a complementary RNA (cRNA) and transcribes the vRNA to produce capped and polyadenylated mRNA molecules (45). Analysis of the replication and transcription activity of the RdRp of human-adapted IAV isolates showed similar steady-state RNA levels at 37°C and 33°C, whereas avian-adapted isolates showed reduced replication at 33°C compared to that at 37°C (82, 83). Importantly, the cold sensitivity of avian IAVs is not cell type dependent or reliant on the introduction of a human-adapted PB2 subunit that restores RdRp dimerization and binding to the essential host factor ANP32A (27, 82, 88). Temperature also has no significant effect on the mutation rate of IAV replication in cell culture (89), suggesting that other properties of the RdRp are responsible for the temperature sensitivity of IAV replication. Interestingly, growth of human-adapted IAV at higher temperatures than the human upper respiratory tract leads to defects in viral growth and in particular a reduction in viral replication and an increase in transcription (48) (Fig. 1A). *In vitro* incubations of purified IAV RdRp at different temperatures showed that the dissociation rate of the RdRp is higher on the cRNA template than on the vRNA template, providing a putative molecular explanation for the reduced viral replication (48). Temperature may also affect the interaction of the IAV RdRp with secondary RNA structures in the viral RNA template, as illustrated by a recent study which showed that RNA duplexes can stall the RdRp and these structures may be less stable at higher temperatures (90) (Fig. 1A).

Passaging of human-adapted IAV, such as A/Ann Arbor/6/60 (H2N2) or A/Leningrad/134/47/57 (H2N2), at temperatures as low as 25°C leads to adaptive mutations in two of the three subunits of viral RdRp as well as the viral nucleoprotein (91). The cold-adapted viruses display attenuated viral growth when they are used to infect cells growing at 37°C. Such attenuated viruses are used in live-attenuated vaccines, as reduced viral replication in the human upper respiratory tract stimulates the induction of a protective immune response while at the same time minimizing the risk of causing clinically significant disease compared to a nonattenuated virus. Research has shown that specific RdRp residues are most important for the cold-adapted growth properties in the Ann Arbor and Leningrad strains (92). When these mutations are introduced into other IAV strains, they also confer a cold-adapted phenotype (93, 94), although it was noted that when they are introduced into an IAV with a PB2 segment of avian origin, the mutations do not lead to restricted growth at physiological temperatures (95), suggesting that the three subunits of the RdRp together define the temperature-dependent replication of influenza viruses, even though it is presently not understood how.

### Coronaviruses.

The CoV genome consists of a single-stranded positive-sense RNA genome of approximately 30 kb in length (96). The long genome is replicated by a viral replication and transcription complex (RTC) that involves at least 16 nonstructural proteins (nsps). To understand how these nsps interact and work in concert to copy the viral genome, numerous temperature-sensitive mutants have been generated (97, 98). Interestingly, the genetic mutations conferring the temperature-sensitive phenotype can be found in nearly any viral protein and they can have vastly different effects, with some abrogating negative-sense RNA synthesis and others positive-sense RNA synthesis (97). Similar to the case with IAV, it is presently not understood what interactions between the viral proteins may underlie the observed cold-adapted phenotypes.

**FIG 2:**
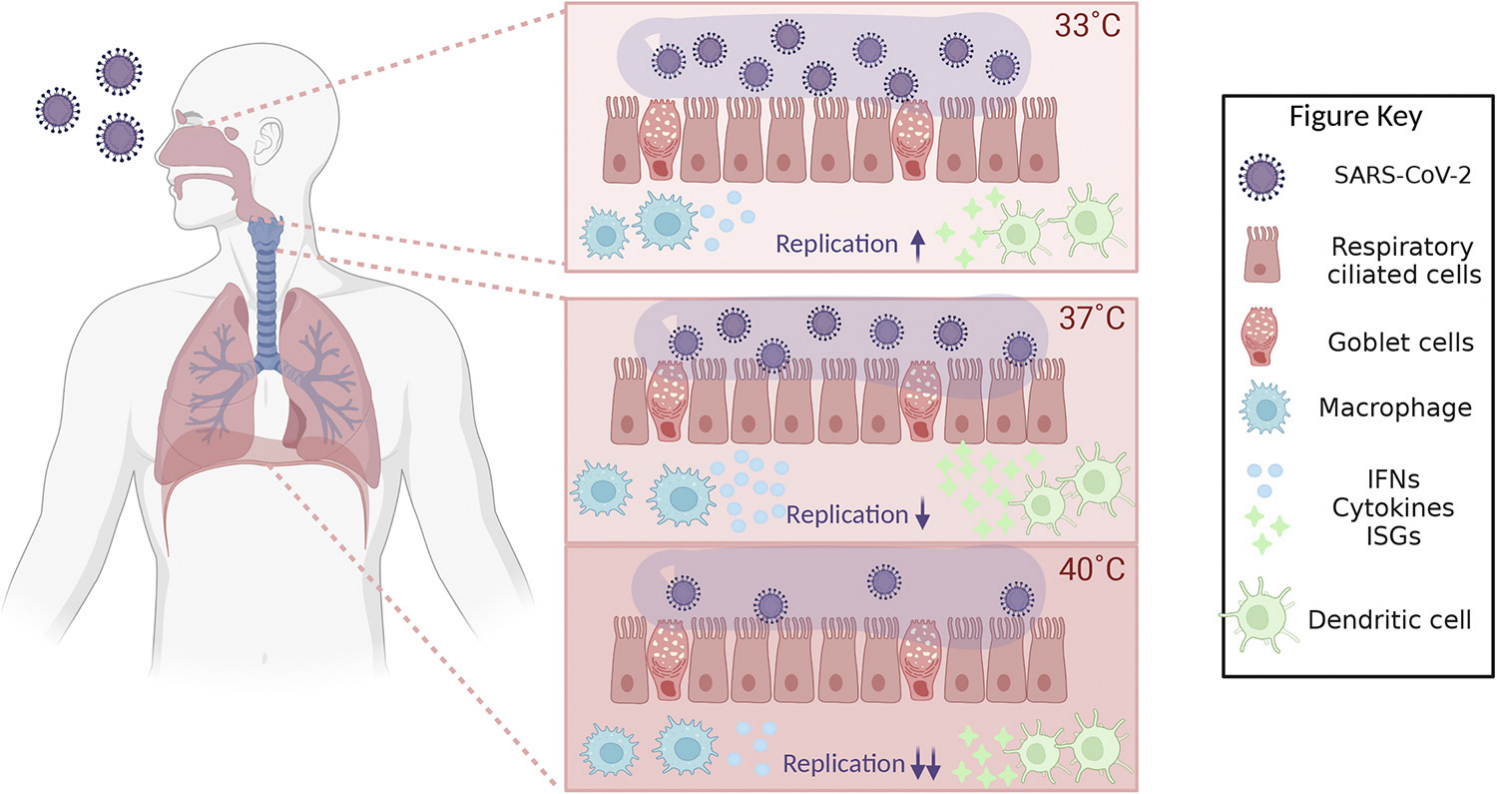
Effect of temperature on SARS-CoV-2 RNA synthesis, SARS-CoV-2 virus titer, and the host innate immune response. Within the human respiratory tract, temperatures vary substantially. SARS-CoV-2 virus efficiently infects cells in the upper respiratory tract, where the temperature is around 33°C. Further down the airways, the temperature slowly increases to 37°C. Additionally, the body temperature can reach >40°C under fever-like conditions. The efficiency of viral replication (indicated with arrows), viral growth (indicated with the number of viral particles depicted), and the host immune responses (indicated with number of IFNs and cytokines depicted) are strongly affected by these different temperatures, resulting in optimal viral replication at 33°C and an optimal immune response at 37°C. ISGs, interferon-stimulated genes. The figure was created using BioRender.

Natural CoV isolates also display different temperature sensitivities. Pandemic SARS-CoV-2 showed a 10-fold-higher replication efficiency at 33°C than at 37°C in hAECs (55) (Fig. 2). An additional increase in temperature to 39°C and 40°C limited the rate of viral RNA synthesis and reduced the viral titer even further (53) (Fig. 2). The preference for growth at 33°C may enhance the ability of SARS-CoV-2 to spread from the upper respiratory tract to the next host. Faster viral growth on hAECs at 33°C than at 37°C has also been observed for seasonal human CoV 229E (99). In contrast, SARS-CoV, which emerged in 2003 and has a higher mortality than SARS-CoV-2, grew to lower viral titers at 33°C and 37°C than SARS-CoV-2, suggesting that it did not evolve an adaptation to a lower temperature to improve its spread in the few months it infected the human population. Similarly, MERS-CoV, which was first reported in 2012 as a zoonotic virus from camels, did not show increased infectivity at 33°C (100). Interestingly, the increased replication of SARS-CoV-2 at 33°C is matched by the increased infectiousness mediated by the viral spike protein, as shown using a pseudovirus containing the SARS-CoV-2 spike (100).

### Rhinovirus.

RVs frequently cause common cold in adults and more severe lung problems in children (101). In addition, RVs cause clinical disease in immunocompromised adults, particularly in those with solid-organ or bone marrow transplants. Most RV isolates replicate more robustly at 33°C than at 37°C (102), and viral RNA accumulates to higher levels at 33 to 34°C than at 37°C (54). However, in contrast to the case with IAV and CoV, RV RdRp activity is not inherently temperature dependent and the rates of RNA synthesis *in vitro* are comparable at 33°C and 37°C (103). This temperature independence is also evident in the epidemiology of RV, which is less seasonal than other respiratory viruses (104). However, when the RdRp activity is analyzed over a broader temperature range (25 to 45°C), such as in a recent single-molecule study, it is observed that the nucleotide addition rate and activation energy of the RV RdRp do dramatically increase as a function of temperature, while the mutation rate of nucleotide incorporation remains constant (105). These findings suggest that other factors contribute to the defined growth of RV at a specific temperature. Indeed, analysis of RV-infected mouse primary airway cells showed that the innate immune response is reduced at 33°C compared to 37°C, enabling RV to grow with fewer restrictions in the nasal cavity than in the lower respiratory tract (54). Similar to the case with CoVs and influenza viruses, passaging of RV at lower temperatures resulted in temperature-sensitive mutations in the viral replication machinery, but how these mutations confer this characteristic is presently not understood.

### Flaviviruses.

Flaviviruses, which include WNV, DENV, and ZIKV, contain a genome that consists of a single-stranded, positive-sense RNA that encodes one large open reading frame (ORF). Intrinsic protease activity leads to cleavage of the viral polyprotein and release of mature proteins, including the viral RdRp. The ORF is flanked by extensive RNA structures that play a key role in the translation and replication of the viral genome. The RNA elements, such as “downstream of AUG region” (DAR) in the ZIKV genome, regulate the transition of the viral genome from a linear to a circular form (Fig. 1B) and undergo structural rearrangements, including melting of the 3′ stem-loop structure (3′ SL), to allow sequences in the two-genome termini to hybridize (106). Only in the circular form can the viral RdRp efficiently bind the 5′ end of the genome (5′ SLA) and transfer to the 3′ end to initiate viral RNA synthesis (107). Mutations that stabilize the stem-loop in the 3′ end of the viral genome reduce viral replication (108). Moreover, a single-nucleotide mutation in DAR is sufficient to limit *de novo* synthesis at 28°C compared to 37°C (51). Additionally, the shift of the virus from a mosquito to a human host resulted in a higher binding activity of human host protein AUF1 p45 to 3′ SL, which influenced the viral RNA structure, eventually resulting in a higher replication rate. However, no such effect was observed on the viral RNA structure in the presence of mosquito AUF1 p45, likely because the mosquito homolog was not highly active at 28°C (50).

In line with the above observations, WNV replication is slower at 28°C than at 37°C, and *Culex* mosquitoes kept at 30°C also displayed increased WNV infection rates, due to a higher replication rate, compared with those at 18°C (109). Moreover, the replication impairment caused by a stabilized stem-loop structure can be partially restored by raising the replication temperature from 28°C to 37°C (50). Similar observations were made for Aedes aegypti mosquitoes infected with DENV-2. These mosquitoes, infected with a low dose of DENV-2 and maintained at 20 to 30°C, showed that infection rates were correlated with the incubation temperature and incubation time (110). In the case of ZIKV, infections of Aedes aegypti mosquitoes reached an infection optimum around 29°C and minimal infection rates at 16°C and 38°C (71). Providing an explanation for the link between ZIKV replication efficiency and temperature, it was recently found that at 20°C, ZIKV RNA synthesis, as measured by double-stranded RNA (dsRNA) formation, is reduced, while other steps in the infection cycle, such as entry, translation, and egress, are not (111). Importantly, this effect of temperature on ZIKV replication can be modulated through mutation, because while the Asian ZIKV lineage was found to replicate slowly at 20°C compared to the optimal temperature, the African ZIKV lineage replicates efficiently and produces similar virus titers at 20°C and 28°C (111).

Analysis of DENV RNA synthesis *in vitro* demonstrated that the viral RdRp synthesizes a maximum amount of product RNA at temperatures between 29.1°C and 31.9°C, although some of this product was double the expected size and likely the result of copy-back RNA synthesis (52) (Fig. 1B). Interestingly, the optimal temperature for the initiation of viral RNA synthesis, which depends on a *de novo* initiation mechanism, was observed at 20°C, whereas the minimal initiation rate was observed at 40°C. It is possible that the structure of the template RNA or the template entry channel of the RdRp adopts a conformation that is optimal for initiation at lower temperatures (52), facilitating efficient replication in mosquitoes.

## EFFECT OF TEMPERATURE ON INNATE IMMUNE RESPONSE

### Respiratory RNA viruses.

The innate immune system detects viral RNA molecules using pattern recognition receptors (PRRs), such as RIG-I-like receptors (RLRs) (112, 113). Upon binding to viral RNA, these PRRs trigger the expression of innate immune genes, including interferon genes (112, 113). Interestingly, temperature was found to directly impact the host response against RNA virus infections. For instance, RV infections of mouse airway cells induce a less efficient type I interferon response at 33°C compared to infections at 37°C (54). In addition, RV is restricted by increased apoptosis and RNase L-dependent degradation of the viral RNA at 37°C in human bronchial epithelial cells (114), explaining why RV can grow to higher titers in the upper respiratory tract than in the lower respiratory tract. Similar differences between infections at 33°C and 37°C have been observed for SARS-CoV-2 in hAECs. Using time-resolved transcriptomic analyses, a stronger induction of both antiviral and proinflammatory responses against SARS-CoV-2 was observed at 37°C than at 33°C (Fig. 2). In particular, a higher expression of chemokines CXCL10 and CXCL11, and PRR RIG-I, was noticed at 37°C (55), suggesting that SARS-CoV-2 growth is less restricted at 33°C, in turn due to a reduced immune response at this temperature.

At temperatures higher than 37°C, the host response is reduced as well (Fig. 2). For example, it was shown that a high temperature (40°C) reduced the production of inflammatory cytokines interleukin 6 (IL-6), IL-1b, and IL-8 when human tracheal epithelial cells were infected with IAV (Fig. 1A) (49). These findings are supported by a recent study in which it was observed that SARS-CoV-2-infected cells show higher beta interferon (IFN-β), IFN-γ, and interferon-stimulated gene expression levels at 37°C than at 40°C (Fig. 2) (53). These reduced responses may exacerbate infections with RNA viruses that spill over and replicate well at higher temperatures, such as emerging avian IAV strains. The molecular mechanism underlying the difference in host response in respiratory epithelial tissues remains to be investigated further (53).

### Vector-borne RNA viruses.

Mosquitoes activate several immune signaling pathways upon viral infection, including the Toll signaling pathway, RNA interference (RNAi), the JAK-STAT pathway, and the Imd pathway (115, 116). Here, temperature also shapes how these responses function (117). In WNV infections of the *Culex* mosquito, a secreted molecule called Vago, which functions similarly to interferons in mammals, is known to activate the JAK/STAT pathway (118). Components of these signaling pathways were optimally expressed at 28°C compared to lower or higher temperatures, suggesting that they may play a role in modulating virus growth (Fig. 1B). Indeed, temperature-dependent regulation of the RNAi pathway was observed during infections with yellow fever virus (YFV), CHIKV (119) and DENV (77). Moreover, this temperature-dependent impairment of the RNAi pathway resulted in increased susceptibility of A. aegypti to CHIKV infection. These observations are in line with a recent transcriptome study of CHIKV-infected mosquitoes at 18°C, 28°C, and 32°C showing a distinct gene expression profile for each temperature, with a lower expression of genes associated with mosquito immune response at 32°C (120). A similar transcriptome analysis was performed for mosquitoes infected with ZIKV to study the temperature-associated variation in gene expression at 20°C, 28°C, and 36°C. Gene expression associated with the Toll signaling pathway and innate immune response was modestly higher at 20°C than at the other two temperatures (121), and viral replication was absent at this 20°C. Overall, these findings suggest that mosquitoes mount a poorer antiviral response at higher temperatures and thus that they are more susceptible to infection and more likely to transmit viruses when local temperatures rise.

In the mammalian host, the type I response was shown to be important in the antiviral response against CHIKV and dengue virus infection (122, 123). However, studies with mice have shown that this response is sensitive to temperature fluctuations during CHIKV infection, demonstrating in particular a reduced response against viral infection at suboptimal temperatures (Fig. 1B). These observations are in line with findings showing that at reduced temperatures, CHIKV replication in mice is increased (124). The exact mechanisms underlying the role of temperature fluctuation in the immune response are not fully understood, but recent studies do demonstrate that it plays a delegate role in host-pathogen interactions and that plenty remains to be explored.

## MIGRATION AND EVOLUTION OF RNA VIRUSES IN RESPONSE TO TEMPERATURE

The observation that the transmission of various vector-borne viruses is limited at lower temperatures and reaches an optimum around 23 to 26°C suggests that climate warming may increase the transmission efficiency of these viruses in temperate regions and decrease the transmission in warmer areas, overall altering the spread of the viruses across the globe (74). In support of this hypothesis, a recent study showed that temperature was an important driver for the introduction and spread of WNV in California in the United States (125). In addition, temperatures changes may impact the parasitic load of vectors and their ability to inhibit or support vector amplification (126). However, temperature-adaptive mutations in strains may change or even compensate for these changing transmission efficiencies. Indeed, following the emergence of WNV in the United States in 1999, new genotypes appeared that spread more efficiently through the mosquito and human populations. Interestingly, one of the first new genotypes studied had a temperature-dependent advantage when transmitted via *Culex* mosquitoes and the transmission of this new strain was shown to increase with rising temperatures (79). Similar, temperature-dependent replication rates of different genotypes have been observed among ZIKV strains with African lineage replicating efficiently at both 20°C and 28°C (111). Since studies have shown that various mutations can support temperature-dependent adaptation of RNA viruses (see “Temperature Impact on Virus Replication, Transcription, and RNA Structure” above) and the short generation times of vectors support adaptation to new environments (127), it seems likely that when temperatures increase for longer periods of time, RNA viruses will adapt to maintain their transmission rates at elevated temperatures and that overall RNA virus spread over the globe will increase.

## TOOLS TO STUDY THE EFFECT OF TEMPERATURE ON RNA VIRUSES AND THEIR INTERACTIONS

Temperature alters the biophysical state of proteins, lipids, and RNA molecules, as well as interactions among proteins and RNA. Historically, temperature-based virus selection was a powerful technique to study host-virus infections, to understand viral replication or transcription dynamics, and to generate live-attenuated vaccines (128, 129). To better understand what these mutations alter in virus-host RNA or protein complexes, standard biochemical tools are used but also high-throughput techniques. One new technique to probe the RNA-protein and protein-protein interactions as a function of temperature is thermal proteome profiling (TPP). It can reveal molecular interaction networks and the stability of the interactions in these networks (130) and can be used to study which links in the interaction network change upon RNA virus infections or between different infections (131). For instance, TPP revealed that during SARS-CoV-2 infection, the virus induces changes in the cell cycle and microtubule and spliceosome complexes (131), highlighting the role of endogenous host protein machinery being utilized by the virus for its survival.

In addition to interaction networks, temperature can strongly affect the folding of secondary RNA structures in the viral genome and the binding of viral or host cell proteins to these structures. Various computational tools are available to study these structures and their interactions as a function of temperature, such as the RNAtips (temperature-induced perturbation of structure) web server (132). Computational approaches have led to interesting insights. A comparison of the folding free energy of RNA structures in avian and human IAV RNA genome showed that the viral genome segments that encode the RdRp subunits of human-adapted IAV have a higher folding free energy than the same segments of avian-adapted IAV (133), suggesting that the secondary structures of avian-adapted IAV are more stable in the human respiratory tract than the structures of human-adapted IAV. Moreover, as avian-adapted IAV adapts to humans, the folding free energy of its genome shifts to higher, less stable values (133). Finally, we can use phylogenomics to study the evolution of a virus population at different temperatures. Such an approach may help us understand the link between virulence and virus population fitness as a function of the temperature (134).

## CONCLUDING REMARKS

Temperature is an important abiotic factor that affects both the host innate immune response and the RNA virus replication machinery. Several decades of research have made it clear that infections with respiratory RNA viruses trigger reduced antiviral responses at the lower temperature of the upper respiratory tract compared to those at the physiological temperature of the lower respiratory tract. This allows RNA viruses to replicate to higher titers and spread more efficiently to the next host. In addition, temperatures above the physiological temperature, such as during the febrile response, reduce innate immune responses and may impair the growth of human-adapted viruses, although they may have a reduced effect on the growth of certain emerging viruses. Importantly, a similar interplay has been observed for arbovirus infections, regardless of whether they are replicating in the vector or mammalian host. In spite of many groundbreaking studies, several open questions remain. For instance, how does temperature impact virulence and pathogenicity, which are partly driven by a dysregulated host immune response and hypercytokinemia (135–137), following zoonotic transmission? And how are virulence and pathogenicity maintained or altered if RNA viruses frequently change between vertebrate and invertebrate hosts and are exposed to different host temperatures, or when they adapt to new temperatures? While it will be difficult to quantify the impact of these different factors on the viral infection cycle as well as the host response to viral infection, it is vital that we gain further insights into these factors in the future and build better models to estimate transmission risks and reduce the impact of spillover events.
